# Nanozymes in Tumor Theranostics

**DOI:** 10.3389/fonc.2021.666017

**Published:** 2021-10-19

**Authors:** Qiulian Ma, Yanfang Liu, Haitao Zhu, Lirong Zhang, Xiang Liao

**Affiliations:** ^1^ Department of Medical Imaging, The Affiliated Hospital of Jiangsu University, Zhenjiang, China; ^2^ School of Medicine, Jiangsu University, Zhenjiang, China; ^3^ Department of Laboratory Medicine, The Affiliated Hospital of Jiangsu University, Zhenjiang, China

**Keywords:** nanozyme, tumor microenvironment, diagnosis, therapy, theranostics

## Abstract

Nanozymes, a new generation of enzyme mimics, have recently attracted great attention. Nanozymes could catalyze chemical reactions as biological enzymes under physiologically mild conditions with higher-efficiency catalytic activities. Moreover, nanozymes could overcome the shortcomings of natural enzymes, such as easy inactivation, high cost, and low yield. With the development of more and more smart and multi-functional nanosystems, nanozymes display great achievement in tumor biology. In this review, we outline the recent advances of nanozymes in tumor and tumor microenvironment diagnosis, therapy, and theranostics.

## Introduction

Despite the great achievement of traditional cancer treatment, such as chemotherapy and immunotherapy, tumors continue to be a major cause of morbidity and mortality. The crosstalk between tumor cells and the tumor microenvironment (TME) is a critical factor for therapy resistance, relapse, and metastasis (1). Therefore, it is important to explore novel strategies to enhance tumor treatment sensitivity by targeting both cancer cells and TME.

Nanomaterials have recently received great interest in enhancing the outcome of cancer therapy, especially nanozymes. Natural enzymes are the proteins or ribonucleic acid (RNA) with highly specific and catalytic ability to their substrates produced by living cells. However, the intrinsic characteristics of natural enzymes, such as storage difficulty, easy deactivation, and high cost, limit their further clinic application (2). With the unexpected discovery of horseradish peroxidase (HRP) activity of Fe_3_O_4_ magnetic nanoparticles (Fe_3_O_4_ MNPs) in 2007, the artificial nanozymes that display similar catalytic mechanism and efficiency to natural enzymes gradually become research hotspots (3). Nanozymes were firstly identified as nanomaterials possessing intrinsic enzyme-like activities (3). Recently, with the development of chemistry and biology, nanozymes are now termed inorganic or organic nanomaterials possessing intrinsic enzyme-like catalytic activities with abundant advantageous properties compared to natural enzymes, such as lower cost, more facile preparation, higher operational ability, and multi-functionalization (4–7).

Based on the rapid development of nanotechnology, the majority of nanoparticles, such as magnetic nanomaterials, cerium oxide nanoparticles (nanoceria), carbon nanotubes (CNTs), graphene oxide (GO), and gold nanoparticles (Au NPs), have demonstrated their intrinsic redox catalytic activities (3, 8–16). Due to the unique properties of nanozymes and the specific characteristics of tumor and TME, deeper and wider applications of nanozymes in tumor diagnosis, therapy, and theranostics are becoming more and more possible. In this review, we firstly briefly summarize the classification of the most common nanozymes and then discuss the promising applications and challenges of nanozymes in the field of tumor theranostics.

## Classification of Nanozymes

**Figure 1:**
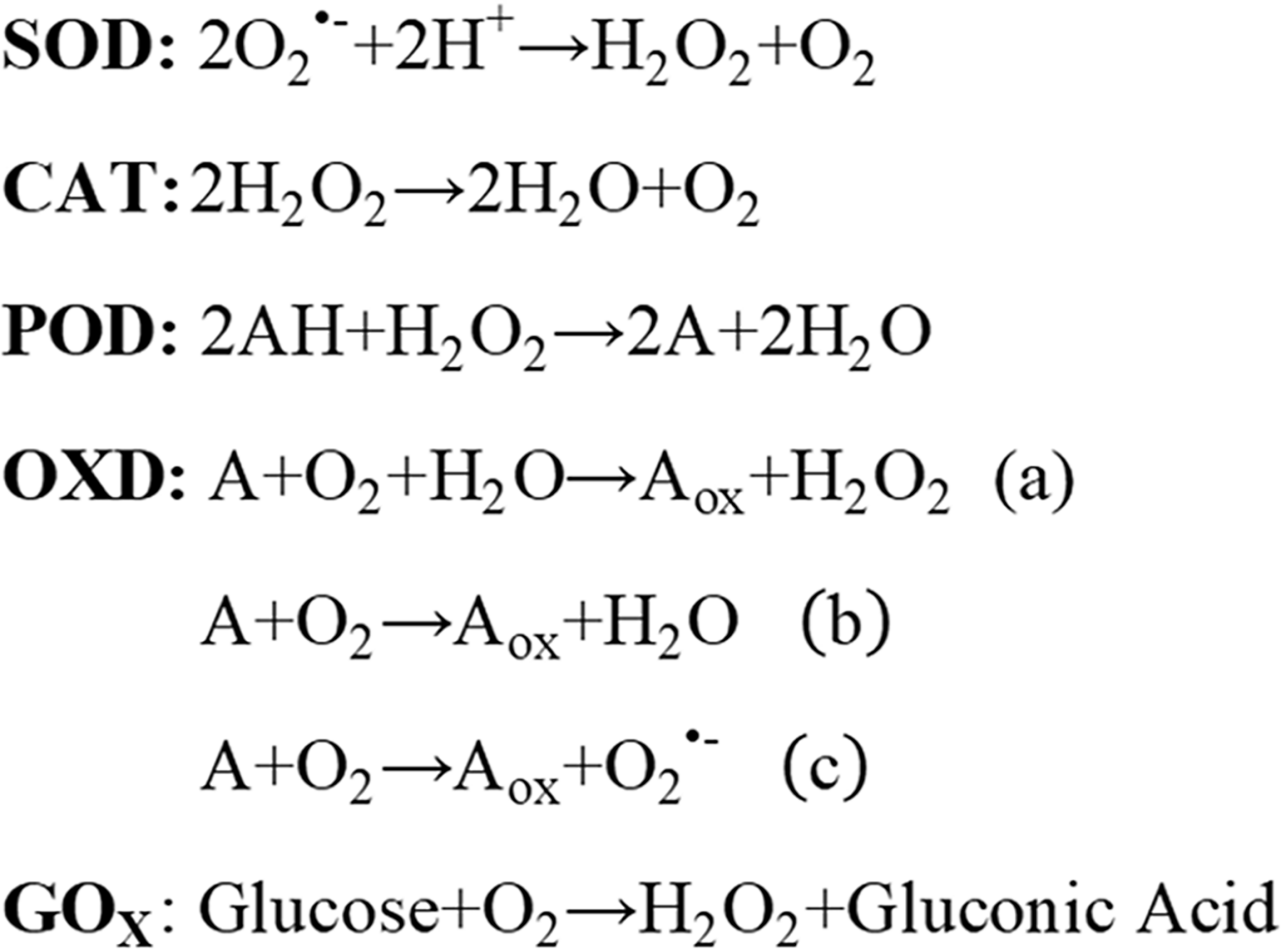
Nanozyme reaction formulas.

Nanozymes mainly include the following subtypes: peroxidase (POD), oxidase (OXD), catalase (CAT), and superoxide dismutase (SOD) (**Figure 1**). More importantly, great efforts must be devoted to the exploration of novel nanozymes. In this section, we discuss only a few parts of representative nanozymes based on their compositions.

### Carbon-Based Nanozymes

Carbon-based nanomaterials, including carbon nanotubes (CNTs), graphene oxide (GO), carbon nanospheres, and carbon nanodots (C-Dots), have been proved as the POD mimic catalytic enzyme (11, 15, 17–23), while fullerene and its derivatives perform the SOD-like activity (4).

Combining the ability of hemin to catalyze various oxidation reactions and the large open surface area and rich surface chemistry of graphene, the nanoplatform-modified hemin onto the surface of graphene through the π−π stacking can serve as POD enzymes and display stable geometric support and efficient molecular loading ability (19, 20).

The carboxyl-modified graphene oxide (GO-COOH) with the intrinsic POD property could catalyze the peroxidase substrate 3,3’,5,5’-tetramethylbenzidine (TMB) in the presence of hydrogen peroxide (H_2_O_2_) (15). The accompanying blue color reaction makes them capable to be developed for a cheaper and more sensitive glucose detection (15).

CNTs can be distinguished as single-wall carbon nanotubes (SWNTs) and multi-wall carbon nanotubes (MWNTs) according to the number of graphene layers (24). SWNTs could catalyze the substrate of TMB, which have been developed to target dsDNA efficiently (11). Moreover, it has been confirmed that the enzymatic activity of carbon nanotubes strongly depended on pH, temperature, and H_2_O_2_ concentration (11).

Based on the superior enzyme activities of nitrogen-doped carbon nanomaterials (N-CNMs), N-doped porous carbon nanospheres (N-PCNSs) possess excellent mimic activities, including OXD-, POD-, CAT-, and POD-like activities (25). These activities are positively correlated with the concentration of N dopant and can also be tunable by pH and temperature (25, 26). Additionally, the B/Fe-doped carbon nanoparticles can also function as POD catalysts (26, 27).

### Metal-Based Nanozymes

With the high glucose conversion ability, gold nanoparticles (Au NPs) have been discovered to perform POD- and OXD-like activities (13, 14). Mesoporous silica nanoparticles (MSN) or bovine serum albumin (BSA) can be assembled on the surface of Au NPs for the detection of glucose or dopamine (DA) by the distinguished GOx- and POD-like activities of Au NPs (28, 29). However, high temperature can result in the poor catalytic performance of Au NPs due to the instability of enzymatic product ABTS^•+^, which can be improved by ionic liquid (30). The stable platinum nanoparticles (Pt NPs) have the ability to scavenge H_2_O_2_, superoxide anion $\left({\text{O}}_{2}^{-}\right)$ , and singlet oxygen (^1^O_2_), simulating CAT-, SOD-, and OXD-like activities. The specific catalytic enzyme activities of Pt NPs are tightly dependent on temperature and pH (31, 32). Under the low pH environment, Pt NPs mostly possess POD-like activity, while Pt NPs exhibit CAT- and SOD-like activities under neutral conditions (31, 32). Moreover, there is a positive correlation between enzyme activities and Pt content. Escapsulating apo-ferritin on the Pt NPs (PtNP@apo-ferritin), this system exhibited more outstanding SOD-like activity and longer-term stability (32, 33).

### Metal Oxide-Based Nanozymes

Nanoceria and iron oxide magnetic nanoparticles (Fe_3_O_4_ MNPs) are the most widely utilized metal oxide catalysts among the metal oxide-based nanomaterials (5, 8–10, 16, 34, 35). Nanoceria exists in a mixed valence state (Ce^3+^and Ce^4+^) (9, 36–38). The ratio of Ce^3+^ and Ce^4+^ determines the catalytic enzyme activity of nanoceria. Nanoceria mainly performs SOD-like activity with a high Ce^3+^/Ce^4+^ ratio, while performing CAT-mimic activity with a low Ce^3+^/Ce^4+^ ratio (7). Moreover, the activity of nanoceria and Fe_3_O_4_ MNPs can be controlled by pH. Under the low pH environment, nanoceria possesses an intrinsic OXD-like activity (9). Fe_3_O_4_ MNPs display a POD-like activity under acid conditions, while showing CAT-like activity in a neutral environment through the decomposed H_2_O_2_ (39–41). The manganic oxide nanoparticles (MnO NPs) behave as the SOD, CAT, and GOx enzymes, inducing the elimination of hydroxyl radical (·OH), maintaining redox homeostasis, and protecting cells from neurotoxin-induced damage (42).

### Metal Chalcogenide Nanozymes

Copper monosulfide (CuS) nanoparticles (CuS NPs) have been demonstrated to perform POD-mimic activity by catalyzing the peroxide substrate 3,3’,5,5’-TMB in the presence of H_2_O_2_ (43–47). Moreover, with the CuS NPs further covered on the graphene, the CuS-graphene nanosheets (CuS-GNSs) possess higher intrinsic POD- and GOx-like activity than CuS or graphene, respectively, which have been employed to detect H_2_O_2_ concentration and monitor the human blood glucose level (44). CuS concave polyhedral superstructures (CuS CPSs) possess superior POD-like activity compared to either the initial formed spherical CuS superstructures or convex CuS microspheres, due to the fact that the concave structures constructed by the thinner nanoplates have a hollow/porous structure that led to a higher surface area (43, 48).

It has been proved that several iron chalcogenides can serve as POD-mimic enzymes. FeS_2_ nanosheets (FeS_2_ NSs) possess the ability to oxidate the peroxide substrate TMB due to the Fe ion located in the active site (49). Simultaneously, the peroxidase activity of FeS_2_ NSs can be tunable by pH and temperature (49). The FeS_2_/SiO_2_ double mesoporous hollow spheres (DMHSs) not only exhibit a more outstanding POD-like activity than both Fe_3_O_4_ NPs and FeS_2_ NSs, but also are more susceptive to the detection of H_2_O_2_ and glutathione (GSH) (50). The sulfur vacancies in magnetic greigite (SVs-Fe3S4) NSs have demonstrated a distinguished POD-mimic activity resulting from the abundant SVs, which have been developed for the colorimetric detection of glucose in human serum (51).

The MoS_2_ nanosheets have been developed for the regulation of oxidation stress due to their intrinsic multi-enzyme-like activities under physiological conditions, including SOD-, CAT-, and POD-mimicking activities (52). MoS_2_ nanosheets can efficiently remove several kinds of reactive oxygen species (ROS) through the Mo^6+^/Mo^4+^ redox couple and accelerate the electron transfer between TMB and H_2_O_2_ (52).

The reason why nanozymes are considered to have enzyme-like catalytic activities is that they own high catalytic activities and can catalyze the same chemical reactions as biological enzymes. In addition, compared to biological enzymes, nanozymes have superior biocompatibility, stability, and targeting ability, and play corresponding catalytic activities in different environments. The application of nanozymes in tumor diagnosis and treatment depends on their closer integration of nanotechnology and biomedicine for the.

## Nanozymes in Tumor Diagnosis, Therapy, and Theranostics

**Figure 2:**
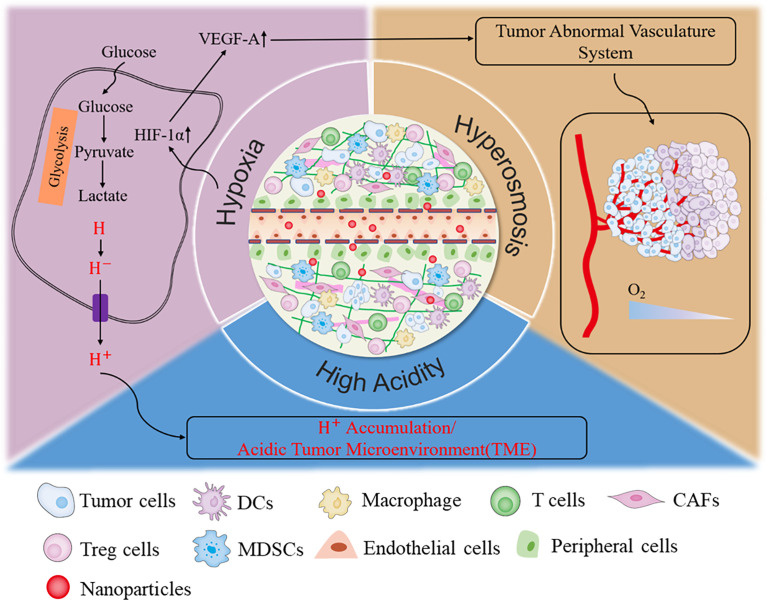
The characteristic of tumor microenvironment.

Solid tumors consist of cancer cells and their living environment, also termed tumor microenvironment (TME). Previously, traditional cancer therapy avenues were mostly focused on cancer cells. Recently, more and more lines of evidence have uncovered that the TME is also critical on tumor malignant behaviors. Therefore, targeting both cancer cells and TME is becoming a promising cancer therapy method. TME includes the various soluble substance and stromal cells, such as fibroblasts, immune and inflammatory cells, glial cells, and other cells, as well as nearby micro-vessels and various biological signal molecules (53, 54). The “interactive cooperation” between stromal cells and cancer cells facilitates the progression of tumor and contributes to the dramatic dynamic changes and the heterogeneity of TME (53). In addition, cancer cells could also remodel the TME, ultimately resulting in the immune escape, metastasis, and even relapse of tumor (55). The characterized metabolism manner, rapid growth, and strong reproduction ability of cancer cells determine their higher demands for oxygen and glucose than normal cells. Cancer cells compete with stromal cells to take advantage of glucose for aerobic glycolysis. Also, abundant lactic acid secreted into the extracellular environment ultimately forms the acid and immunosuppressed TME (56–58). The broken balance between oxygen consumption and supply resulted in the messy growth and irregular distribution of tumor vasculature systems, which, in turn, eventually enhanced the degree of permanent or temporary hypoxia and further increased the osmotic pressure of TME (59). Therefore, low pH, hypoxia, excessive H_2_O_2_ and GSH, high osmotic pressure, and immunosuppressive microenvironment are the outstanding hallmarks of solid tumors (**Figure 2**) (53, 56, 58). These characteristics are mutually causal, finally contributing to the rapid progression of tumor. Targeting and normalizing TME seem to be a new and effective method for tumor diagnosis and treatment. Recently, more and more nanozymes have been constructed to target the diagnosis and treatment of TME.

### Nanozymes in Tumor Cells and Tumor Microenvironment Target Diagnosis

Nanotechnology-based tumor target diagnosis and therapy include passive target and active target. Active target greatly relies on the recognition of the specific receptors overexpressed on cancer cells and the ligand-directed binding on the surface of nanosystems (60). Loaded with special markers on the cancer cell surface, such as transferrin, growth factors, peptides, folate, antibodies, or antibody fragments, nanozyme systems not only recognize tumor more sensitively, but also result in drug delivery more specifically (61). The nanozymes modified with folic acid can actively target the folic acid receptors on the cancer cell surface and further can serve as oxidants to promote cancer cell death (9, 17). Porous platinum nanoparticles on graphene oxide (Pt NPs/GO) can function as peroxidase mimetics. which enable them to detect cancer cells by the color reaction of TMB (62). Furthermore, by loading folic acid on the Pt NPs/GO, this nanosystem can distinguish a total of 125 cancer cells more broadly than naked-eye observation (62). Prostate-specific antigen (PSA), a special tumor biomarker, can be attached by the immune complexes based on the intrinsic POD-like activity of GO, and then the PSA concentration could be directly detected with the colorimetric reaction (63). Ultra-small gold nanoclusters (Au NCs) can serve as POD-like catalysts for disproportionation and decomposition of H_2_O_2_, which make them sensitive probes for tumor imaging *in vivo* (64). The multifunctional protease nanosensor constructed by Au NCS not only can determine whether the tissue is cancerous through the catalyzed reaction of Au NCS according to the color reaction, but also is non-toxic and can be completely eliminated by liver and kidney excretion (64). Furthermore, the magneto-ferritin nanoparticles (M-HFn) are composed of iron oxide and heavy-chain ferritin (HFn) shell (65). Due to the ability of targeting transferrin receptor 1 (TfR1) overexpressed on cancer cell surface and the color reaction in tumor site resulting from the POD-like activity of the iron oxide core that could catalyze the abundant H_2_O_2_ in TME, M-HFn could visualize cancer tissues sensitively and specifically (65). Similarly, HFn-N-PCNSs-3 can also specifically identify the cancer cells and effectively reduce the tumor volume dependent on the special binding of TfR1 on cancer cell surface and the multi-enzyme mimic activities of N-PCNSs-3 (25). Besides, angiopep-2, a specific ligand of lipoprotein related protein-1 (LRP1), anchored on the surface of Au NPs can penetrate through the blood–brain barrier (BBB) and further actively target glioma cancer cells (66, 67).

Magnetic resonance imaging (MRI) contrast agents based on the ROS-stimulated responses, such as superoxide ions, H_2_O_2_, and hydroxyl radicals, have been promising tumor diagnostic and imaging markers due to their extensive accumulation and persistent presence in TME (68–70). Prussian Blue nanoparticles (PBNPs, KFe^3+^[Fe^2+^(CN)_6_]) perform CAT-like activities under the neutral pH condition (71). The core Fe^3+^ with water coordination can form paramagnetic oxygen bubbles, which are conducive to shorten the MRI T1-weighted image (T1WI) relaxation time and then enhance the MRI contrast (72). Based on the previous pioneering work of PBNPs, SPIO@GCS/acryl/biotin-CAT/SOD-gel (SGC), a dual-enzyme-loaded multifunctional hybrid nanogel probe, has been developed to strengthen the ultrasound imaging and the imaging contrast of T2WI (73). In recent years, the PB@Au core-satellite nanoparticles (CSNPs) have been constructed to explore multiple diagnostic and therapeutic strategies of tumors (72). CSNPs can achieve dual-model imaging due to the PB NPs that acted as MRI T1WI contrast agents and the enhanced computed tomography (CT) imaging efficiency by AuNPs (72). Besides, the MnO NPs exposed to the superoxide radicals could enhance the MRI signal and simultaneously treat the catalytic-induced tumor progression due to their intrinsic SOD-mimic ability (74). Moreover, the CAT-like nanoparticles are gradually utilized as coupling or contrast agents of ultrasound (US) and MRI owing to the enhanced catalyzed H_2_O_2_ into O_2_ molecules (71–73).

Early diagnosis of tumor makes it possible to obtain outstanding tumor clearance and satisfactory clinical prognosis by local treatment. Nowadays, the early detection of tumor mainly depends on the blood tumor markers and imaging manifestations. However, the extremely low abscission rate of early tumor markers or the lack of specificity of imaging findings limits the accuracy and sensitivity of early tumor detection in clinical. The emergence of nanozymes provides new ideas and methods for the early diagnosis of tumor and the visualization of tumor tissues, which greatly improve the specificity and sensitivity of early diagnosis of tumor.

### Nanozymes in Synergistic Tumor Therapy

Nanozymes can achieve anti-tumor effects by improving TME. For example, the highly ordered MnO_2_@PtCo nanoflowers are developed as a ROS generation nanoplatform for tumor therapy by targeting the hypoxia and the acidic pH of TME (75, 76). Cooperating with the OXD-like activity of PtCo and the CAT-like activity of MnO_2_, the MnO_2_@PtCo nanozymes not only could supply O_2_ to overcome the hypoxic TME, but also catalyze ROS formation, which further induces the admirable tumor apoptosis (75). Similarly, the DMSN-Au-Fe_3_O_4_ composited nanoplatforms could make the TME-responsive tumor vanish owing to the GOx-mimic activity of Au NPs and the POD-like activity of the Fe_3_O_4_ nanoparticles. The DMSN-Au-Fe_3_O_4_ nanozymes are capable of catalyzing β-D-glucose oxidated into gluconic acid and subsequently produce high-toxic hydroxyl radicals for tumor regression (41). Based on the abundant GSH detained in TME, pyrite nanozymes and FeS_2_ with ultrahigh H_2_O_2_ affinity promote the glutathione oxidation due to their OXD-like activity and the generation of ^•^OH by their POD-like activity, resulting in the ferroptosis and apoptosis of tumor cells consequently (35). Recently, a novel nanosystem, polyethylene glycol (PEG)-ylated iron manganese silicate nanoparticles (IMSN) loaded with TGF-β inhibitor (TI) (IMSN-PEG-TI), has also been constructed to regulate the tumor immune microenvironment and advance the tumor therapeutic modality through the intrinsic POD- and CAT-like activities of IMSN nanozymes under the acidic TME (58).

Additionally, nanozymes can synergistically enhance the anti-tumor effects of tumor therapy avenues that deeply depend on the oxygen level, such as photodynamic therapy (PDT), photothermal therapy (PTT), sonodynamic therapy (SDT), radiotherapy (RT), and chemotherapy (1, 77). Moreover, consumption of O_2_ and tumor vasoconstriction can further exacerbate hypoxia and limit the efficiency of the above tumor therapies, which finally form a positive feedback (78–81). Nanozymes are used more and more widely in enhancing the efficiency of these therapies.

#### Nanozymes in Synergistic Phototherapy

Phototherapy relies on light radiation to induce the death of cancer cells, including PDT and PTT. PDT firstly transforms light energy to the surrounding O_2_ and then produces a high concentration of cytotoxicity of ROS to further oxidize biomacromolecules and induces their dysfunction (77, 82–84). PTT induces the death of tumor cells depending on the local thermal damage (77, 84, 85). Although the photosensitizers and photothermal agents could enhance therapy efficiency and reduce the side effect of PDT and PTT under near-infrared (NIR) laser irradiation, they convert to excited single states and then return to the ground states by collisions between surrounding molecules. Accompanied by the increased kinetic energy, they consequently result in the heating of the surrounding microenvironment (77, 85).

Although the metal-organic frameworks (MOFs) assembled with photosensitizers can induce the death of tumor cells by the conversion of oxygen into ^1^O_2_, the efficiency of PDT is still limited owing to the hypoxia of the TME (80, 81, 86). The novel Pt nanozymes have been placed on the photosensitizers integrated with MOFs to break the limitation of hypoxia. This nanoplatform not only possesses higher stability, but also performs CAT-like activity leading to the additional ^1^O_2_ formation and further enhancing the efficiency of PDT (81).

CAT-mimicking Pt NPs are sandwiched into the dual-nanozyme-engineered porphyrin metal organic frameworks (PCN); furthermore, the outer GOx-mimicking Au NPs coordinate with folic acid (Pt@P-Au-FA) (87). The Pt@P-Au-FA NPs can enhance O_2_ generation by catalyzing H_2_O_2_, which further enhances PDT efficiency. What is more, Au NPs strengthen the depletion of glucose and the self-produced H_2_O_2_ serve as substrates of Pt NPs, cooperating with glucose depletion-induced starving therapy and achieving remarkable anti-tumor effects (87).

**Figure 3:**
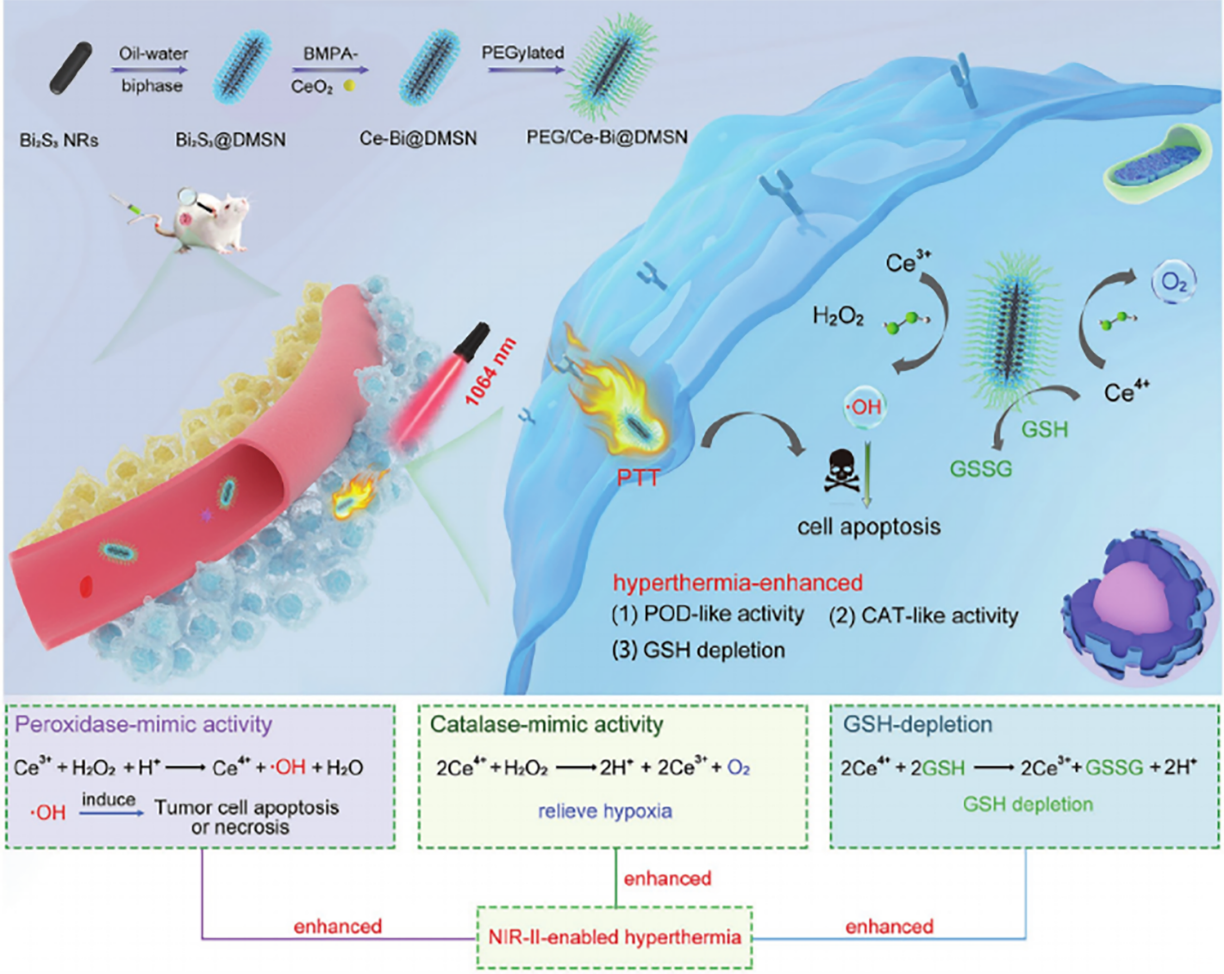
Schematic illustration of the PEG/Ce-Bi@DMSN nanozymes enhancing the efficiency of PTT. Reproduced with permission (88). Copyright 2020, Wiley-VCH.

The nanozyme PEG/Ce-Bi@DMSN is constructed by dendritic mesoporous silica coated with uniform Bi_2_S_3_ nanorods (Bi_2_S_3_@DMSN) and further by ultrasmall ceria placed into the large mesopores of Bi_2_S_3_@DMSN, which possesses dual mimic catalytic activities (including POD- and CAT-mimic activities) under primary acidic TME resulting in elevated oxidative stress and relieved hypoxia (88) (**Figure 3**). Additionally, PEG/Ce-Bi@DMSN allowed the enhanced GSH consumption to be overexpressed in TME. The NIR laser irradiation could strengthen the catalytic activities and GSH depletion of PEG/Ce-Bi@DMSN nanozymes, which further synergistically enhance the tumor ablation effect of PTT (88).

The platinum-doped Prussian blue (PtPB) nanozyme was developed to improve the photothermal property in a large wavelength range during the process of PTT (89). On the other hand, the PtPB nanozyme is endowed with superior CAT and SOD-like catalytic activities by Pt doped with PB nanotubes, which contributed to the relieved inflammation caused by PTT, along with significant tumor inhibition (89).

#### Nanozymes in Synergistic Sonodynamic Therapy

Ultrasound (US)-triggered sonodynamic therapy (SDT) consisting of a low-intensity ultrasound and a chemotherapeutic agent (sonosensitizer) is a promising alternative tumor therapeutic modality (90–93). US not only is more accessible and noninvasive in reaching deep-seated tumor tissues, but also can activate sonosensitizers to produce toxic ROS molecules for tumor eradication (90, 91, 94). However, the therapeutic efficiency of SDT is still restricted by severe hypoxia in TME to a great extent (90, 95).

The hollow Pt-CuS Janus can overcome the hypoxia environment due to the mimetic enzyme activity of Pt that decomposes the endogenous overexpressed H_2_O_2_ into O_2_ (47). The hollow Pt-CuS Janus has superior photothermal performance, which not only elevates the Pt enzyme activity for O_2_ production, but also augments the SDT-induced tumor cell death by higher ROS level simultaneously (47). Hence, the synergistic efficiency of PTT and the catalysis-improved SDT can achieve complete tumor elimination.

The nanoprobe (CDP@HP-T), constructed by Pt-embedded hollow polydopamine (P@HP) nanoparticle, co-loaded with doxorubicin (DOX) and chlorine e6 (Ce6) and further modified with the mitochondrial-targeting molecule triphenyl phosphonium (TPP), can be used to achieve enhanced combination therapy of chemotherapy and SDT for tumors (94). As a pH-responsive nanoprobe, the CDP@HP-T could realize the abundant O_2_ generation and alleviate the hypoxia of tumor sites responsible for the CAT-like activity of Pt and endogenous overexpressed H2O2 under weakly acidic TME, which further enhances the efficacy of SDT (94). Concomitantly, with DOX and TPP, this nanoprobe could achieve tumor eradication by inhibiting cellular DNA replication, further enhancing the combined therapeutic efficacy of chemotherapy and SDT (94).

**Figure 4:**
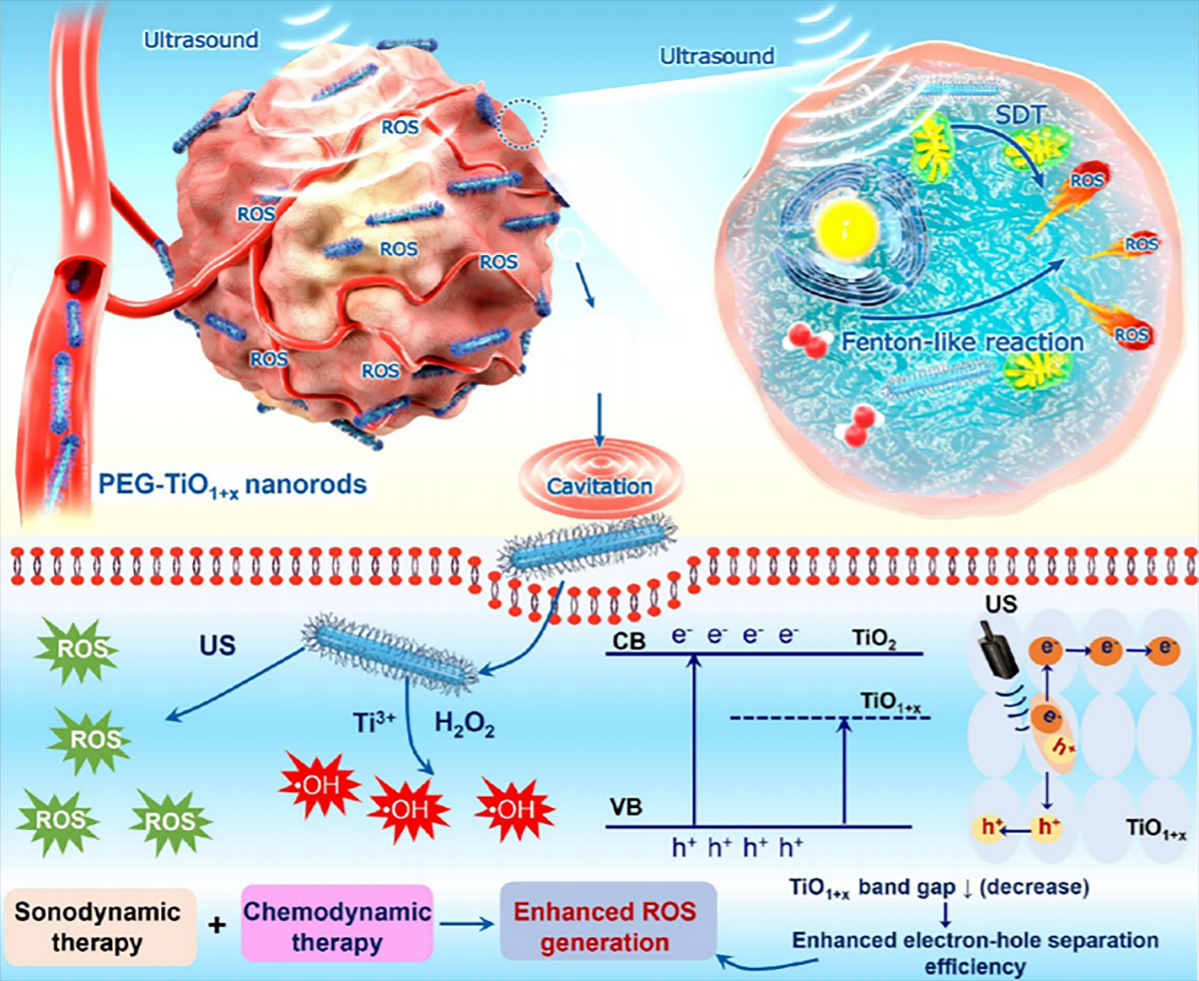
Schematic illustration of PEG-TiO_1+x_ NRs that served as sonosensitizers in the synergistic chemotherapy and SDT. Reproduced with permission (96). Copyright 2020, American Chemical Society.

Analogously, the ultrafine titanium monoxide (TiO_1+x_) nanorods modified with PEG (PEG-TiO_1+x_ NRs) enable higher tumor elimination outcome in synergistic chemotherapy and SDT (**Figure 4**) (96). The TiO_1+x_ NRs possess POD-like activity for the decomposition of H_2_O_2_ in TME (96). Notably, the PEG-TiO_1+x_ NRs could generate superior US-induced ROS due to the oxygen-deficient structures within TiO. On the other hand, the PEG-TiO_1+x_ NRs could serve as Fenton-like agents for ROS generation in the presence of Ti^3+^ (96).

#### Nanozymes in Synergistic Radiotherapy

Radiotherapy (RT) has been widely used as the first-line treatment modality of various solid tumors in clinics (97–101). However, the TME complex leads to the ultimate resistance to RT and even the recurrence and metastasis of tumors (98). Normalizing the TME to enhance the effectiveness of RT, to improve hypoxia and increase intratumoral oxygen concentration, and to further promote radiation-induced DNA damage is one of the most common strategies.

Several nanomaterials are designed to enhance tumor radiation sensitivity and attenuate hypoxia by catalyzing the generation of O_2_ (102, 103). The high reactivity, stability, and specificity of the albumin complex and MnO_2_ NPs (A-MnO_2_ NPs) towards H_2_O_2_ could simultaneously modulate hypoxia and acidosis TME with regulated pH (102). Furthermore, A-MnO_2_ NPs could normalize tumor blood vessels by the downregulated hypoxia-inducible factor-1α (HIF-1α) and vascular endothelial growth factor (VEGF) (102). Taking advantage of the engineered multifunctional A-MnO_2_ NPs, the tumor response to radiation can be enhanced significantly (102).

Based on the perfect RT responsiveness of MnO_2_, gold and manganese dioxide (Au@MnO_2_) core-shell nanoparticles coated with PEG formed Au@MnO_2_-PEG (104). Au@MnO_2_-PEG, using the Au core, functioned as a RT sensitizer and MnO_2_ shell as CAT mimics that mediate the decomposed H_2_O_2_ could not only overcome tumor hypoxia but also enhance the tumor sensitivity to RT (75, 104). More importantly, the Au@MnO_2_-PEG displays more satisfactory tumor inhibition than the outcome of Au-PEG or MnO_2_-PEG and has good biocompatibility and biosecurity (104).

#### Nanozymes in Synergistic Chemotherapy

Chemotherapy, as the most commonly applied cancer treatment modality, induces cancer cell death partly through regulating the formation of ROS (105). Abundant nanozymes loading chemotherapy drugs have been applied in tumor treatment depending on enhancing the generation of ROS (106).

Iron oxide nanoparticles (IONPs), with the POD-like activity, could decompose H_2_O_2_ into hydroxyl radicals under acidic or neutral conditions (73). The super-paramagnetic iron oxide nanoparticles (SPION) with inherent POD-like activity are proved to reduce H_2_O_2_ in human mesenchymal stem cells (hMSCs) in a dose-dependent manner, and further promote cell proliferation and growth (107). SPION can also be degraded in lysozymes and produce iron ions, which further accelerates the process of cell cycle (106). In addition, the combination of SPION with β-lapachone, an anticancer drug, significantly enhances the intracellular ROS levels and tumor-killing efficiency in non-small cell lung cancers (NSCLCs) (106).

Hollow Ru@CeO2 yolk shell nanozymes (Ru@CeO2 YSNs) loaded with anti-tumor drug ruthenium complex (RBT) and resveratrol (Res), and then modified with PEG, formed the Ru@CeO2-RBT/Res-PEG nanozyme system (108). Ru@CeO2-RBT/Res-DPEG could achieve oxygen supply *in situ* and enhance the anti-tumor responses of both chemotherapy and PDT. Moreover, it can also limit the metastasis and recurrence of tumors (108).

#### Nanozyme Systems for Tumor Theranostics

Based on the development of nanotechnology, more and more multi-functional nanozyme platforms are designed, with the ability of multi-model therapy, multi-model imaging, or simultaneously realizing tumor diagnosis and therapy.

The novel PtFe@Fe_3_O_4_ nanozyme, with outstanding POD- and CAT-like activities in the acid TME, could overcome the hypoxia in tumor and enhance the effects of PDT. Moreover, PtFe@Fe_3_O_4_ could be used as MRI T1WI negative contrast agents (41, 109). The biomimetic hybrid nanozyme (rMGB), integrated with GOx and MnO_2_, could realize the self-catalytic reaction products under TME stimulation, resulting in enhanced O_2_ generation and improving the efficiency of starvation therapy and PDT (110). Also, rMGB could be used as the MRI T1WI contrast agents.

Based on the POD-like and ROS-regulated activity of Au NPs under an acid environment, the carbon–gold hybrid (OMCAPs@rBSA-FA@IR780) nanoprobes not only reveal excellent tumor-targeting imaging ability, but also offer outstanding tumor therapeutic performance (111). Besides, the Au_2_Pt-PEG-Ce6 nanoplatform was developed through Ce6 linked to Au_2_Pt nanozymes covalently (112). Contributing to the photosensitive characteristics of Ce6 and the dual CAT- and POD-like activities simultaneously of Au_2_Pt nanozymes, this nanosystem not only can relieve tumor hypoxia with O_2_ generation but also enhance the efficiency of PDT and chemotherapy with the produced ∙OH (112). Moreover, due to high-Z elements of Au and Pt, Au_2_Pt-PEG-Ce6 can be possible imaging contrast agents of CT (112, 113).

With single-atom Ru incorporated into the Mn_3_[Co(CN)_6_]_2_ MOF framework, followed by the biocompatible poly-vinylpyrrolidone (PVP) polymer further encapsulating organic ligand, metal ions, and photosensitized Ce6, the self-assembled single-atom enzyme (OxgeMCC-rSAE) was constructed (114). As Ru served as an endogenous oxygen-generating single-atom catalytic site, OxgeMCC-rSAE can degrade H_2_O_2_ to generate oxygen, which further enhances the generation of ROS, ultimately enhancing PTT-induced cancer cell death (114). Meanwhile, due to the higher loading of the photosensitizer Ce6, the nanoparticles can selectively aggregate and be visualized in the tumor area by MRI (114).

## Conclusion and Prospects

Since the hallmark ferromagnetic nanoparticles proved to be of use as POD natural enzymes in 2007, nanozymes have attracted unprecedented attention and applications, especially in oncology. Although nanozymes have achieved excellent progress in many areas, there are still many problems that cannot be ignored. Currently, most of the present nanozymes mainly focus on the activity of oxidoreductase and hydrolase activities, but the other enzyme activities such as transferase and lyase are still poorly understood. Therefore, it is necessary to explore new nanozyme materials and study their catalytic properties in depth. In addition, the catalytic mechanism of nanozymes is diverse and regulated by various factors. Moreover, different nanozymes may have a synergistic effect in the anti-tumor process. Therefore, it is necessary to establish completeness for different types of nanozyme catalytic systems. The current catalytic efficiency of nanozymes makes it hard to reach the level of natural enzyme *in vivo*, and their activities are still limited due to the complicated TME. Besides, the poor substrate selectivity of nanozymes persists. Modification of certain specific molecules with nanozymes may solve the problem and may improve the substrate specificity and target the tumor more sensitively and specifically. In addition, studies of nanozymes in tumor theranostics are still in the primary stage. The inherent toxicity and clearance rate of the materials also limit their wide applications. Moreover, various nanozymes have their own unique advantages and shortcomings. Therefore, constructing a nanosystem with good biocompatibility, high targeting efficiency, and multiple functions would be a crucial task.

With the continuous development of nanoscale science and technology, nanozymes show superior versatility, operability, and applicability, thus paving the way for new principles and technologies in disease diagnosis and treatment as well as efficient and precise new nanodrug applications in the biomedical field (**Table 1**).

**Table 1:** Nanozyme Classification and Applications.

Nanozyme system	Mimetic activities	Applications	References
**Carbon-Based**			
SWNTs	POD	Drug delivery; Human SNP DNA detection	(11, 24)
GO	POD	Tumor visual detection	(63)
GO-COOH	GOx; POD	Glucose detection	(15)
GFH	POD	Tumor detection	(17)
C-Dots	POD	Glucose detection	(22)
HFn-N-CNMs-3	POD; OXD (acidic pH values)	Tumor catalytic therapy	(25)
	SOD; CAT (neutral pH values)	Anti-oxidant therapy	(25)
**Metal-Based**			
Au NPs	GOx	Self-limiting nanomedicine; Biomedical probe	(14, 111)
EMSN-AuNPs	GOx; POD	Self-activated cascade catalysis	(28)
AuNCs	POD	Tumor detection	(64)
BSA-AuNCs	POD	A dual fluorometric and colorimetric sensor for dopamine	(29)
Au/SiO_2_ nanocomposites	POD	Realizing high-temperature catalytic reactions	(30)
AuNCs-NH_2_	CAT	Enhancing PDT efficiency	(83)
Carbon-gold hybrid nanoprobes	CAT	Real-time imaging, enhancing PTT and PDT efficiency	(111)
Au_2_Pt-PEG-Ce6	CAT; POD	Synergistic chemotherapy and phototherapy	(112)
Pt NPs	CAT	Enhancing RT efficiency	(113)
Pt NPs/GO	POD	Tumor detection	(62)
PtPB	CAT; SOD	Enhancing PTT efficiency	(89)
Pt-MOFs hybrid system	CAT	Enhancing PDT efficiency	(81)
Pt-Carbon nanozyme	CAT	Enhancing PDT and PTT efficiency	(84)
P@Pt@P−Au−FA	CAT; GOx	Synergistic starving-like therapy and PDT	(87)
Pd@Pt-T790	CAT	Enhancing SDT efficiency; anti-bacterial infection	(92, 93)
CDP@HP-T	CAT	Synergistic chemotherapy and SDT	(94)
AFeNPs	Fenton reaction	Enhancing MRI contrast and chemotherapy effects	(105)
Fe@BC	POD	Anti-bacterial infection	(27)
Rh-PEG NDs	CAT	Anti-inflammation and anti-tumor	(46)
OxgeMCC-r SAE	CAT	Enhancing PDT efficiency	(114)
IMSN-PEG-TI	POD; CAT	Anti-tumor	(58)
**Metal Oxide-Based**			
Fe_3_O_4_ MNPs/IONPs	POD (acidic pH values)	Detection of organophosphorus pesticide and nerve agent	(3, 16, 39)
	CAT (neutral pH values)	Anti-oxidant	(39)
PtFe@Fe_3_O_4_	POD; CAT	Synergistic tumor catalytic therapy and PTT	(109)
DMSN-Au-Fe_3_O_4_ NPs	POD; GOx	Anti-tumor	(41)
Nanoceria	SOD (neutral pH values)	Against radiation damage, oxidative stress and inflammation	(9, 10, 38)
		Fluorogenic detection of cancer	
Folate-conjugated Nanoceria	OXD (acidic pH values)	Cancer detection	(9)
PEG-CNPS	SOD	Radical scavenger with tunable redox chemistry	(36)
A-MnO_2_ NPs	CAT	Modulating TME and enhancing RT responses	(102)
MnO NPs	SOD	Enhancing MRI contrast	(74)
Au@MnO_2_-PEG	CAT	Enhanced RT *via* improving the tumor oxygenation	(104)
Mn_3_O_4_ NPs	SOD; CAT; GPx	Anti-inflammation	(76)
MnO_2_@PtCo	OXD; CAT	Anti-tumor	(75)
rMGB	CAT	Enhancing starvation and PDT against hypoxic tumor	(110)
Ru@CeO_2_-RBT/Res-DPEG	CAT	Enhancing dual chemotherapy combined with PTT	(108)
PEG-TiO_1+x_ NRs	POD	Enhancing dual chemotherapy combined with SDT	(96)
**Metal Chalcogenide**			
CuS-GNSs	POD; GOx	Detection of H_2_O_2_ and human serum glucose level	(44)
Hollow Pt-CuS Janus	POD	Synergistic PTT and SDT	(47)
DMHSs-FeS_2_/SiO_2_	POD	Detection of H_2_O_2_ and GSH for anti-tumor	(50)
SVs-Fe_3_S_4_	POD	Detection of human serum glucose level	(51)
FeS_2_	OXD; POD	Anti-tumor	(35)
PEG/Ce-Bi@DMSN	POD; CAT	Synergistic tumor catalytic therapy and PTT	(88)

## Author Contributions

QM and YL wrote the draft of the manuscript. HZ and LZ contributed to the conception of the work and organized the structure of the manuscript. XL performed the revision. All authors contributed to the article and approved the submitted version.

## Funding

This work was supported by grants from the National Natural Science Foundation of China (grant number 82071984), the Young Medical Talents of Jiangsu (grant number QNRC2016833), the Six Talent Peaks Project of Jiangsu Province (grant number WSW-039), the Six for One Project of Jiangsu Province (grant number LGY2018093), the Project of Postgraduate Research & Practice Innovation Program of Jiangsu Province (SJCX19_0577), and the Social Development Foundation of Zhenjiang City (grant number SH2021071).

## Conflict of Interest

The authors declare that the research was conducted in the absence of any commercial or financial relationships that could be construed as a potential conflict of interest.

## Publisher’s Note

All claims expressed in this article are solely those of the authors and do not necessarily represent those of their affiliated organizations, or those of the publisher, the editors and the reviewers. Any product that may be evaluated in this article, or claim that may be made by its manufacturer, is not guaranteed or endorsed by the publisher.
